# Evaluation of the Influence of Coating and Coating Composition on the Sorption Properties of Freeze-Dried Carrot Bars

**DOI:** 10.3390/molecules30081716

**Published:** 2025-04-11

**Authors:** Agnieszka Ciurzyńska, Magdalena Karwacka, Monika Janowicz, Sabina Galus

**Affiliations:** Department of Food Engineering and Process Management, Institute of Food Sciences, Warsaw University of Life Sciences—SGGW, Nowoursynowska Str., 159c, 02-776 Warsaw, Poland; magdalena_karwacka@sggw.edu.pl (M.K.); monika_janowicz@sggw.edu.pl (M.J.); sabina_galus@sggw.edu.pl (S.G.)

**Keywords:** gelatin, freeze-dried sample, coating, sorption isotherms, vegetable bar, FT-IR

## Abstract

This study aimed to investigate the effect of dip coating and the composition of the applied coating on the structure and sorption properties of freeze-dried carrot bars. The scope of the work included preparing freeze-dried carrot bars, coating them with coatings of different gelatin concentrations, and then analysing the sorption properties based on sorption isotherms. Additionally, the structure was assessed based on porosity, shrinkage, and microscopic observations. Water activity and dry matter content were also measured. Analysis of the obtained results showed that coating caused a significant increase in water activity and a decrease in the dry matter content of freeze-dried carrot bars. There was also a decrease in porosity and volume compared to the control sample, which was confirmed by microscopic analysis. The study of sorption kinetics showed that the coatings limited the hygroscopicity of the samples, reducing the dynamics of moisture adsorption and accelerating the stabilisation of water content. The best model describing the sorption isotherms was the Peleg model, and the isotherms themselves were classified as type IIb according to the Blahovec and Yanniotis classification. The composition of the coating significantly affects the structure and selected physical properties of the bars. FT-IR analysis did not show any significant changes in the bars’ chemical structure.

## 1. Introduction

Growing consumer awareness of the negative effects of using plastic in food storage has led to an increased demand for other, more natural methods of food preservation. To meet these requirements, scientists have taken a closer look at using edible by-products from food processing or renewable biopolymers [1]. These raw materials have been used to produce edible coatings, which can extend the shelf life of food by limiting gas exchange [2] and provide other benefits compared to traditional packaging materials [3].

Edible coatings are thin layers of material that serve as primary packaging while allowing the consumption of packaged food [4]. They provide a barrier to chemical, biological, and physical changes [5]. The coatings are applied by spraying, dipping, or electro-spraying, which allows for obtaining a uniform layer of appropriate thickness [6].

Edible coatings are made of substances with film-forming properties, which are dissolved in a solvent (usually water, alcohol or their mixtures) during synthesis [7]. Substances improving or modifying the material’s functionality are also added, such as plasticisers, e.g., glycerol, sorbitol, polyols, etc. [8]. In the 1960s, research began using gelatin to produce edible coatings [9]. Gelatin coatings are valued for their transparency, good barrier, and mechanical properties, and the possibility of their production by extrusion and casting methods [10]. Gelatin-based edible films are gaining popularity as an alternative to traditional plastic packaging due to their biodegradability, low production costs, and favourable mechanical and barrier properties, such as flexibility, tensile strength, and the ability to limit water vapour and oxygen sorption, which helps to extend the shelf life of food products [11,12,13,14,15]. Dipping is the most popular film-forming method. It consists of dipping the food in the coating solution. There are three steps in the coating process: 1. immersion of food in the coating solution, 2. draining the food from the solution, and 3. drying the coating. This method is indicated for food products with uneven surfaces [16].

Depending on whether a given dried product has been subjected to the homogenisation process or is a whole dried product, it has different sorption properties. Knowing these features in the case of freeze-dried products is one of the most important aspects that directly impact the optimisation of future drying, storage stability, and the final quality of the dried product. Knowledge of sorption properties is necessary to determine water activity, durability level, and critical moisture content, especially for products whose quality deteriorates under the influence of moisture. To know them, the kinetics of water vapour adsorption should be examined, and sorption isotherms should be determined [17]. Sorption isotherms determine the equilibrium relationship between the amount of water adsorbed by a unit of mass of a food product and water activity at a constant temperature and constant total pressure. The final drying point of the material, corresponding to the desired water activity, can also be determined from the isotherm course. Knowledge of this point, with the known critical humidity of the product at a given temperature, makes it possible to decide on the maximum humidity of the drying air in convection dryers or the maximum pressure in dryers operating under reduced pressure and freeze-drying [18].

According to the International Union of Pure and Applied Chemistry (IU-PAC) recommendations, sorption isotherms are divided into several types [19]. In the case of food, type II or type III isotherms are most often observed. The difference in their shapes is based on the interactions between water (sorbate) and food (sorbent). In the case of type II, sorbate–sorbent interactions are stronger than sorbate–sorbate interactions. For type III isotherms, the situation is reversed. Type I isotherms are very rare in food products because of the shape, which lacks the possibility of multilayer sorption [20]. In some cases, it is challenging to distinguish type II from type III isotherms and the moment of transition from one to the other. To avoid the above uncertainty, Blahovec and Yanniotis [21] proposed a methodology based on the transformation of the isotherm to the form a_w_/Me = f(Me). Analysing the behaviour of the curves in the low water activity range, they noticed that the initial curves deflect downwards, and then the deflection changes in the opposite direction. This property is the basis for classifying the isotherm as type II or III. Type II isotherms show an upward deflection, while type III shows a downward deflection. Type I isotherms in this equation are linear [20]. Many mathematical models can be used to determine the sorption isotherm, but the most popular are the BET, Oswin, Halsey, GAB, Lewicki, and Peleg models [18,22].

In the available literature, one can most often find research results on using coatings before drying fruits and vegetables. In the case of freeze-dried products, coating before freeze-drying causes the coating to lose its barrier properties, and a porous structure similar to the entire freeze-dried product is obtained. Therefore, coating food after freeze-drying may be an interesting solution.

## 2. Results

### 2.1. The Influence of Coating Composition on Selected Properties of Freeze-Dried Carrot Bars

**Figure 1:**
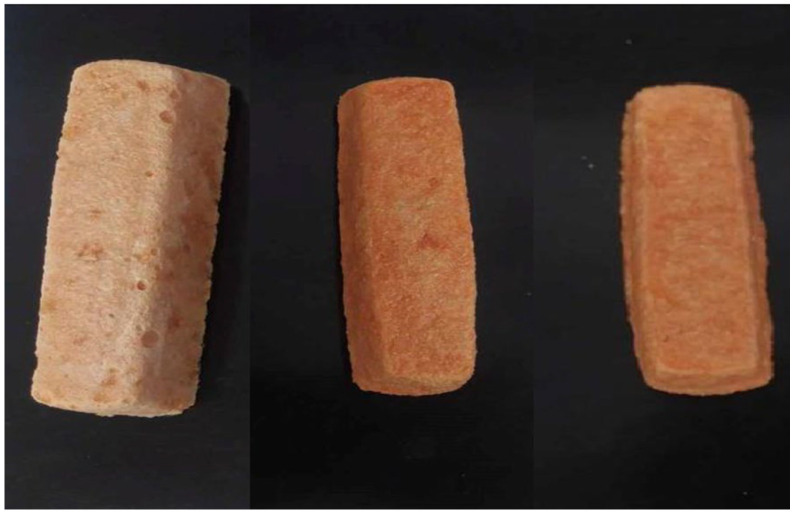
Photos of freeze-dried carrot bars coated with 8% and 12% pork gelatin. From left: control sample, bar coated with 12% gelatin, bar coated with 8% gelatin.

Selected physical properties were tested on dip-coated freeze-dried carrot bars prepared based on available literature and experimental studies. Coatings with 8% and 12% gelatin were used (Figure 1). Uncoated bars were used as a control sample.

#### 2.1.1. Dry Matter Content

**Figure 2:**
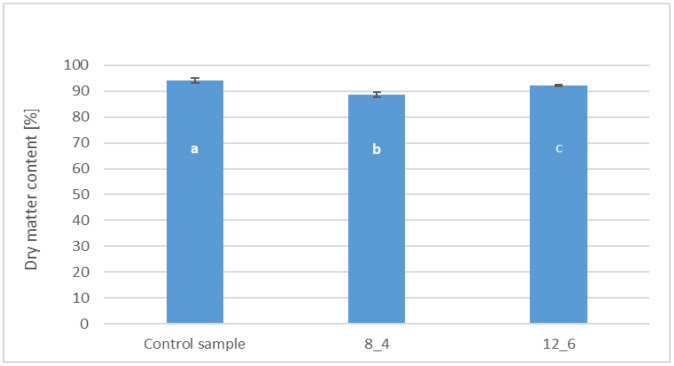
The effect of coating and coating composition on the dry matter content of freeze-dried carrot bars, uncoated [Control sample] and coated with 8% [8_4] and 12% [12_6] gelatin. The columns with different letters “a–c” are significantly different (*p* < 0.05). Designations in Table 4.

The dry matter content in bars coated with 8% and 12% gelatin was, on average, 88.64% and 92.22%, respectively (Figure 2). In the control sample, uncoated, the average dry matter content reached 94.27%. All samples differed from each other statistically significantly in the tested parameter.

#### 2.1.2. Water Activity

**Figure 3:**
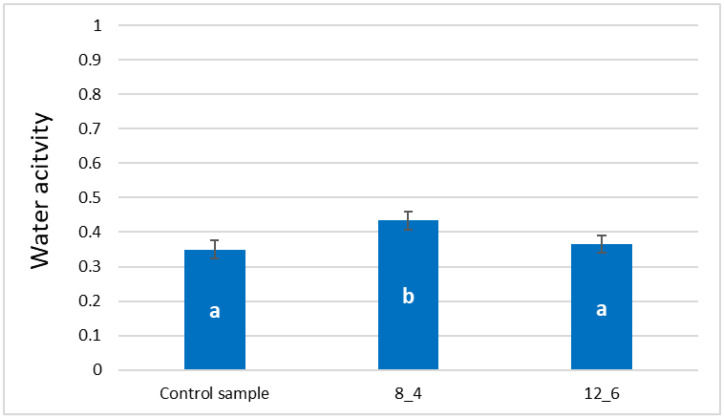
Effect of coating and coating composition on water activity of freeze-dried carrot bars, uncoated [Control sample] and coated with 8% [8_4] and 12% [12_6] gelatin. The columns with different letters “a–b” are significantly different (*p* < 0.05). Designations in Table 4.

The application of a coating of freeze-dried vegetable bars caused an increase in the water activity (a_w_) of the samples. Still, no statistically significant differences were found between the control sample and the one coated with a 12% gelatin coating (Figure 3). The control sample was characterised by the lowest average water activity at the level of 0.35. The value marked for the sample coated with a 12% gelatin coating was slightly higher (0.37). The water activity of the samples coated with an 8% gelatin coating was, on average, 0.43 and was statistically significant (i.e., the highest).

#### 2.1.3. Porosity and Shrinkage

**Figure 4:**
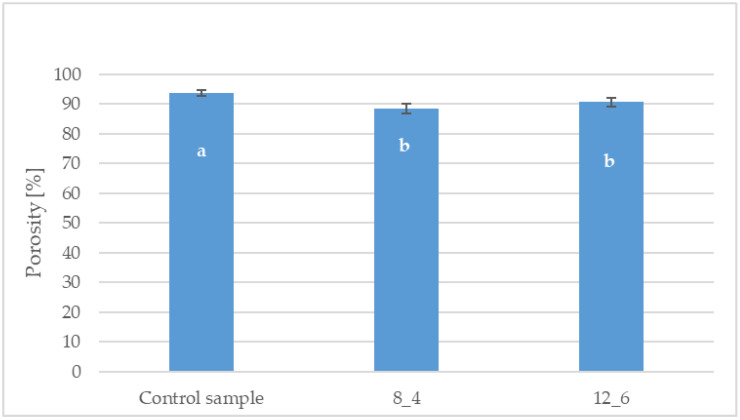
Effect of coating and coating composition on the porosity of carrot bars, uncoated [Control sample] and coated with 8% [8_4] and 12% [12_6] gelatin. The columns with different letters “a–b” are significantly different (*p* < 0.05). Designations in Table 4.

The porosity of the bars, both coated and uncoated, exceeded 88% (Figure 4). The highest value was observed for the control sample, which had a porosity of 93%. Coating the freeze-dried bars resulted in a statistically significant decrease in the tested parameter compared to the control sample. Statistical analysis showed no significant differences in porosity between the coated samples depending on the coating composition.

**Figure 5:**
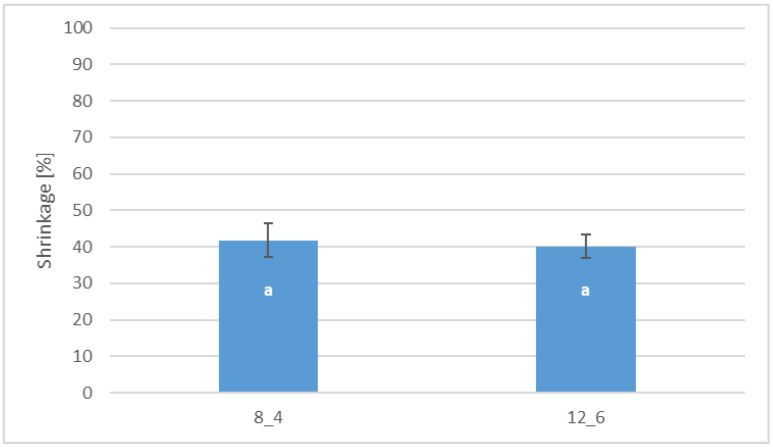
Effect of coating and coating composition on the shrinkage of freeze-dried carrot bars coated with 8% [8_4] and 12% [12_6] gelatin. The columns with different letters “a” are significantly different (*p* < 0.05). Designations in Table 4.

Coating by immersion, especially in the case of freeze-dried products, which are characterised by a delicate and porous structure, causes shrinkage of the dried product at the level of 40% compared to the control sample (Figure 5). There was no statistically significant difference between the coated samples depending on the gelatin concentration.

#### 2.1.4. Sorption Isotherms

Table 1 presents the determined variables of the individual models and the calculated fit coefficients to describe the RMS, MRE, SEE, R^2^, and RSS sorption isotherms for each of them. By comparing these coefficients, it was possible to select the best-fit model to describe the sorption isotherms of freeze-dried carrot bars. The selection criteria were based on the selection of the lowest values of the RMS (percentage root mean square error), MRE (percentage mean relative error), RSS (sum of residual squares) and SEE (standard error of estimate) coefficients and, at the same time, the highest coefficient of determination R^2^ [18,22].

It was found that the RMS values were very high regardless of the type of samples. The RMS coefficient of the control sample ranged from 41.85% (Oswin’s model) to 167.35% (Hasley’s model). Very similar results were obtained by the GAB (42.1%), Lewicki (44.19%) and Peleg (47.73%) models. In the case of samples coated with 8% gelatin and 12% gelatin, the lowest RMS coefficient occurred in the case of the Peleg model at the levels of 39.36% and 42.36%, respectively. The remaining models, in the case of coated samples, showed RMS results above 100%. A similar distribution of results was observed for the MRE, RSS and SEE coefficients, where the Peleg model showed the lowest values in relation to the coated samples. In contrast, for the control sample, the GAB and Peleg models showed the lowest values. The values of the coefficient of determination R^2^ for the control sample were the highest for the GAB and Peleg models at the level of 0.997, while for the coated samples the highest value was shown by the Peleg model at the level of 0.999.

**Figure 6:**
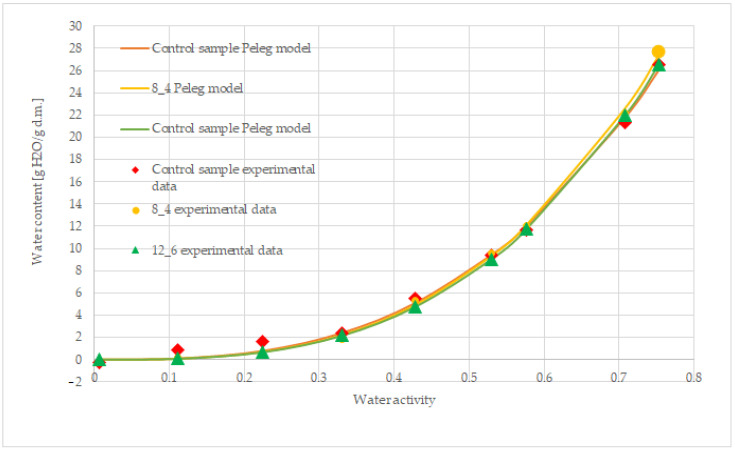
Fitting the Peleg model to describe the sorption isotherms of freeze-dried carrot bars, uncoated [control] and coated with 8% and 12% gelatin.

To sum up all the results, the Peleg model is the best fitting model for describing the sorption isotherms of all the samples. The fit of this model to the description of the obtained sorption isotherms is shown in Figure 6.

Freeze-dried carrot bars show type II according to the classification of Brunauer et al. [23]. The procedure and equation described by Blahovec and Yanniotis [21] were used to classify sorption isotherms. Nonlinear regression allowed the classification parameters for freeze-dried bars to be obtained, which are presented in Table 2. The obtained values of the determined parameters for the freeze-dried carrot bars of the control sample and those coated with 8% and 12% gelatin indicate that their isotherms can be classified as type IIb with differences in hygroscopicity and adsorption dynamics.

#### 2.1.5. Infrared Spectroscopic Analysis (FTIR)

**Figure 7:**
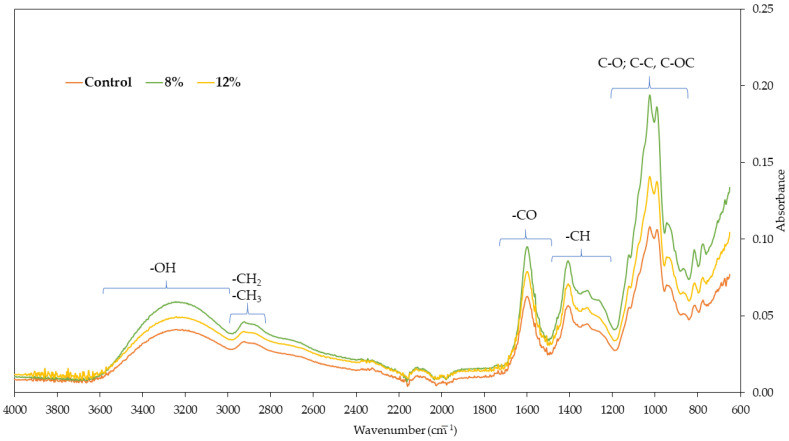
The Fourier transform infrared spectra of freeze-dried carrot bars.

Fourier transform infrared spectra of freeze-dried carrot bars are shown in Figure 7. Spectroscopy of different types of bars visible on the graph assumes spectra in the wave number range from 650 to 400 cm^−1^. All spectra show the leading characteristic bands associated with carrots. The first range of wave numbers, from 3656 to 2980 cm^−1^, indicates the presence of -OH groups, as water molecules cause vibrations in this area, indicating hydrogen bonds between components. The highest absorbance value in this range was observed for carrot bars coated with 8% gelatin coating, which is in line with the highest water activity value (Figure 3). The peak value at approximately 3265 cm^−1^ indicates the presence of -NH groups. The characteristic spectrum in the absorbance range of 2900 cm^−1^ provides information about the presence of -CH_2_ and -CH_3_ groups. Vibrations in the 1490–1800 cm^−1^ range indicate the presence of C=O bonds. For the bars tested, the highest absorbance was observed at a wavelength of 1595 cm^−1^. The spectrum regions in the 1170–1480 cm^−1^ range indicate the presence of -CH groups and bonds characteristic of methyl groups.

#### 2.1.6. Structure

**Figure 8:**
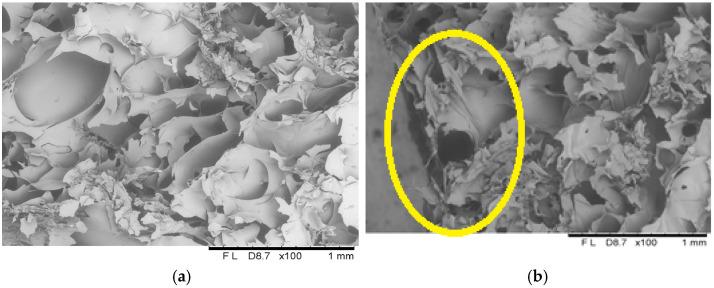
The internal structure of the centre (**a**) and surface (**b**) of a freeze-dried vegetable bar without coating [Control sample]. Yellow circle identify locations where microstructural differences are particularly pronounced.

**Figure 9:**
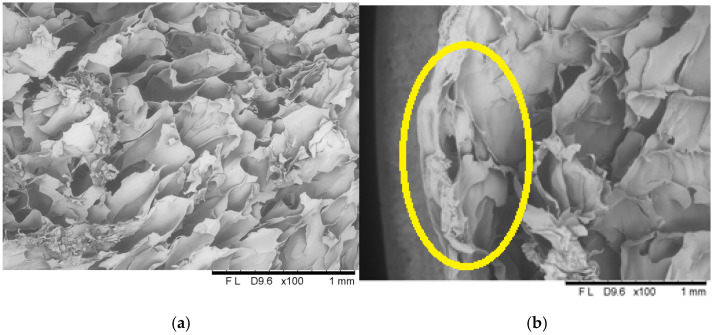
The internal structure of the centre (**a**) and surface (**b**) of a freeze-dried vegetable bar coated with 8% [8_4] gelatin. Yellow circle identify locations where microstructural differences are particularly pronounced.

**Figure 10:**
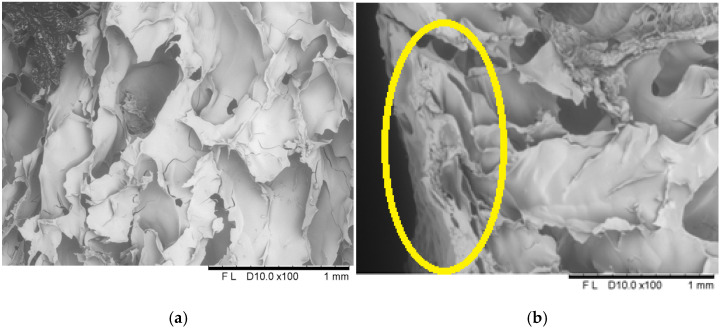
The internal structure of the centre (**a**) and surface (**b**) of a freeze-dried vegetable bar coated with 12% [12_6] gelatin. Yellow circle identify locations where microstructural differences are particularly pronounced.

The microscopic analyses allowed obtaining images showing the microstructure of freeze-dried vegetable bars (Figure 8, Figure 9 and Figure 10). Based on the microscopic images, it can be stated that all the tested samples have homologous morphological features indicating a distinct porous structure, which correlates with the results of the porosity parameter assessment (Figure 4). In the control sample, which included freeze-dried vegetable bars without edible coating, open pores were observed distributed evenly over the entire surface of the sample (Figure 8). The structure of the tested samples was dominated by pores of irregular shapes, which is the result of the manufacturing methodology used, which includes homogenisation and mixing processes.

Applying the dip coating method in coating solutions containing 8% and 12% gelatin (Figure 9 and Figure 10) resulted in a continuous coating that adhered well to the sample surface. The coating showed a tight connection with the bar surface.

## 3. Discussion

The water content in food products is one of the essential criteria determining their quality, nutritional value, and storage suitability. The higher the percentage of water in a given product, the lower the amount of valuable nutrients—proteins, fats, and carbohydrates. In addition, with the increase in water content, the possibility of microorganisms developing in food products increases, and consequently—their suitability for long-term storage without proper technological processing decreases [24]. Water is removed from the product during the drying process, and a dry substance (total dry mass content) is obtained [25].

The highest dry matter content was observed in the control sample, uncoated, followed by bars coated with a 12% addition of gelatin. The lowest result was recorded for samples coated with a coating containing 8% gelatin. Despite differences in the dry matter content, all tested samples were characterised by a dry matter content exceeding 85%, which is typical for freeze-dried products, the basic raw material of which is carrot. In the study by Ignaczak et al. [26] on different drying methods for carrots, the dry matter content of freeze-dried samples was 93.6–95.8%. These results are similar to those presented in this paper and confirm the effectiveness of freeze-drying to remove water from the product, which translates into durability and suitability for consumption. The differences between the dry matter content of the coated samples and the control sample can be attributed to the adhesion of the wet coating to the bar surface layer. The dip coating process introduces a significant amount of moisture into the product and the coating solution, which is not completely removed even during subsequent convective drying. This is confirmed by the obtained water activity results, indicating limited effectiveness of moisture elimination in the coated samples. Similar results were obtained by Ciurzyńska et al. [27] in previous work about the effect of coating with the use of coating and edible films with broth addition of freeze-dried vegetable bars.

In the 1970s, a research team led by Labuza [28] developed the so-called food stability map, which shows the relationship between water activity and the course and kinetics of various processes and reactions. This map takes into account the influence of water activity on factors important for food stability, such as microbial growth, enzymatic activity, the rate of non-enzymatic browning reactions, and the oxidation of fats and dyes. In later studies, Rahman [29] enriched the stability map by introducing relationships related to changes in the mechanical properties of food. The minimum values of water activity (a_w_) necessary for developing different groups of microorganisms are 0.9 for most bacteria, 0.88–0.6 for yeasts, and 0.8–0.6 for moulds. It is generally accepted that developing microorganisms in food with water activity below 0.6 is impossible. The water content in food products allows for the identification of three main water activity ranges: products with high water content are characterised by water activity from 1.00 to 0.90; foods with medium water content range from 0.90 to 0.55; and products with low water content have water activity below 0.55 [30].

The water activity of samples coated with 8% gelatin was significantly the highest, confirming the dry matter content test results. At the time of immersion coating, the bar absorbs water from the coating-forming solution, which, as was found during the analysis of the dry matter content results, is not fully removable even with subsequent drying in a convection dryer. Similar results were obtained by Ciurzyńska et al. [27] for coatings and edible films. The above results are typical for freeze-dried carrot products [26]. All samples showed water activity lower than 0.6, which means they can be considered microbiologically stable and safe [30].

Freeze-dried materials are usually characterised by low shrinkage, high porosity, and preservation of the cellular structure of the raw material, and the drying process does not cause significant changes in their structure [31]. A high porosity coefficient is a desirable feature, as it allows for better rehydration capacity, positively affects sensory properties and reduces the product’s final weight [32]. It was shown that the porosity of the bars, both coated and uncoated, exceeded 88%. Coating the freeze-dried bars significantly reduced their porosity compared to the control sample. The obtained results of the porosity of freeze-dried bars coated with gelatin-based coatings are also typical for freeze-dried products. Krokida et al. [33] obtained an average porosity of freeze-dried carrots in the 70 to 95% range, depending on the freeze-drying temperature used. Ciurzyńska et al. [27] also obtained decreased porosity for freeze-dried vegetable bars after coating. The shrinkage of freeze-dried bars due to coating was at the level of 40%. Coating carrot bars with gelatin-added coatings positively reduces shrinkage by effectively retaining air bubbles in the product, which is confirmed by the high porosity results. On the other hand, the higher shrinkage of the sample coated with 8% gelatin confirms the lower porosity value of this bar due to water penetration from the coating solution with a higher water content than the 12% gelatin coating. Lenart and Piotrowski [34] investigated the effect of coating osmotically dehydrated apples and showed that the coated samples obtained less shrinkage after convective drying than the uncoated samples.

Sorption isotherms describe the relationship between water content and water activity in a given material. Due to the complexity of the chemical composition and structure of food products, selecting the best model describing sorption behaviour can be very difficult [35]. The sigmoidal shape of sorption isotherms was observed for freeze-dried carrot bars, coated and uncoated. The equilibrium moisture content of these samples in the water activity range of 0–0.35 showed lower values, while after exceeding this value, it increased rapidly. A similar course was characteristic for the isotherms in the studies of Kędzierska and Pałacha [18], who analysed the effect of temperature on the sorption isotherms of freeze-dried carrots, and in the studies of Ciurzyńska et al. [22] on freeze-dried multi-vegetable bars, as well as Jakubczyk and Jaskulska [36] on freeze-dried vegetable soups. At low and medium values of water activity, the moisture content increased linearly with the increase in water activity, while at high values of water activity, the water content increased rapidly, which was associated with capillary condensation. Additionally, it can be concluded from the results of the studies of Jakubczyk and Jaskulska [36] that the highly porous structure of the bars at higher water activity contributed to filling the pores with absorbed water, and a further increase in water activity could lead to a rapid increase in the moisture content in the sample. The material could not bind water due to the limited amount of solid matrix.

In the case of the most frequently observed type II isotherms, Blahovec and Yanniotis [21] distinguished three subtypes, IIa, IIb and IIc, based on the Blahovec and Yanniotis [21] isotherm equation and the share of its terms in the total value of sorption on primary and secondary sites. If the coefficients of both types are comparable, the isotherm is classified as subtype IIa. In the case of type IIb and IIc isotherms, the coefficients related to sorption on primary and secondary sites dominate. Based on the best-fit parameters obtained from the description using the isotherm equation, several secondary parameters are calculated (the most important of them are D10, Rfi and X4). The first derivative of this function at the water activity point a_w_ = 0 is D10. The ratio of the first derivative of the a_w_/w − a_w_ graph in the final values to the first derivative in the initial values (a_w_ = 0) is designated as Rfi. X4 is the equation constant [20,36]. The sorption isotherms of the sample with the X4 parameter higher than 0.1, a positive Rfi value, and a positive D10 value can be classified as type IIb, i.e., sigmoidal with dominant capillary condensation, for which a rapid increase in humidity at higher water activities is typical. Based on the determined parameters, freeze-dried carrot bars were classified as type IIb with different hygroscopicity and adsorption dynamics. The control sample shows the highest sorption capacity at lower water activities, as evidenced by the highest D10 coefficient among all samples, with the Rfi index suggesting moderate capillary condensation. The sample coated with 8% gelatin shows the lowest rate of moisture increase at lower water activities but a very high Rfi index of 34.359, indicating the dominance of capillary condensation at high a_w_. The 12% sample has a high D10 coefficient, suggesting significant hygroscopicity at the beginning, and the Rfi index indicates the dominance of capillary condensation. The control sample shows the best stability at low water activity, while the addition of 8% gelatin significantly changes the sorption dynamics, reducing hygroscopicity at lower a_w_ but increasing the capillary condensation capacity at higher a_w_. The sample coated with 12% gelatin retains moderate hygroscopic and condensation properties, which makes it the most universal. Similar results were obtained by Jakubczyk and Jaskólska [36] in studies on freeze-dried vegetable soups and Ciurzyńska et al. [22] on freeze-dried multi-vegetable bars, where, in both cases, it was possible to assign the sorption isotherms of vegetable-based products to type II isotherms according to the Brunauer et al. classification [23].

The models analysed for their fit to the description of the obtained sorption isotherms were the GAB model [37], Hasley [22], Peleg [38], Lewicki [39], and Oswin [40]. For the description of sorption isotherm curves, Peleg’s model was chosen as the best. Due to the very low parameter C (Table 1) at the level of 0.16, the GAB model was rejected. In the context of sorption isotherms, the parameter C in the GAB model refers to the strength of intermolecular interactions, particularly the relationship between the binding energy of water molecules in the monolayer and the condensation energy of water in the upper layers. This condition is a physical criterion that determines the correctness of applying the GAB model to describe the sorption isotherms. If C is not within the range of 5.67 ≤ C ≤ ∞, it means that the GAB model is not suitable for the analysed material or the experimental conditions do not meet the model’s assumptions [41].

Fourier transform infrared spectroscopy (FT-IR) is an analytical technique for identifying chemical composition and internal molecular bonds. It allows for the evaluation of interactions between the components of the tested material [42]. The most intense spectrum for all tested samples is 850–1200 cm^−1^, indicating the presence of C-O bonds, with a peak around 1020 cm^−1^ indicating C-O-C bonds [43]. The spectral area under 900 cm^−1^ provides information regarding conformational changes in tested material and corresponds to the crystalline areas [44]. The most intense spectrum for all tested samples is 850–1200 cm^−1^, indicating the presence of C-O bonds, with a peak around 1020 cm^−1^ indicating C-O-C bonds [43]. The spectral area under 900 cm^−1^ provides information regarding conformational changes in tested material and corresponds to the crystalline areas [44]. Similar results were obtained by Kaur et al. [45] for black carrot pomace powder.

Microscopic cross-sectional analyses clearly showed high porosity present in the entire volume of the samples. Analogous results have been described in the literature, including by Ciurzyńska et al. [22], who analysed the microstructure of freeze-dried fruit bars, and by Karwacka et al. [46], who studied freeze-dried snacks based on frozen vegetable by-products and apple pomace. Additionally, scanning observations allowed for the identification of changes in the structure of the surface layer. Due to the absorption of the film-forming solution, this layer was partially deformed and collapsed, resulting in a decrease in its porosity and an increase in density, which influenced the appearance of shrinkage compared to the control sample. In the case of samples coated with 8% gelatin solution, changes in the surface layer are more pronounced; cell collapse is visible at a greater, more significant surface depth, which translates into a higher level of shrinkage and lower porosity compared to samples coated with 12% gelatin.

## 4. Materials and Methods

### 4.1. Research Material

The research material consisted of freeze-dried carrot bars covered with coatings containing different pork gelatin contents, and the control sample was an uncoated bar. The bars were prepared based on the recipe by Marczak [47] (Table 3).

### 4.2. Preparation of Freeze-Dried Vegetable Bars

The carrots were cut into cubes, blanched in boiling water for 1 min, and then cooled in cold water. The prepared raw material was ground with the addition of salt using a BOSCH MSM817180 blender (BSH Sprzęt Gospodarstwa Domowego Sp. Z o.o., Warsaw, Poland) until a uniform mass was obtained. Then, sodium alginate (Agnex, Warsaw, Poland) and calcium lactate (Agnex, Poland), previously dissolved in a small amount of water, were added. These substances were added to obtain the appropriate structure. The obtained gel was poured into silicone moulds from Tescoma (Katowice, Poland) with dimensions of 14 × 10 × 2.5 cm. After cooling at room temperature, the gel was frozen for 2 h in an Irinox freezer (Irinox S.p.A., Treviso, Italy) to reach a temperature of about −40 °C and then placed on the shelves of a Christ ALPHA 1-4 LSC plus freeze dryer (Martin Christ GmbH, Ostrode am Harz, Germany). During the freeze-drying process, the pressure was maintained at a constant level of 63 Pa, and the shelf temperature was 30 °C for 72 h.

### 4.3. Preparation of Edible Coatings

Edible coatings were prepared based on the modified methodology developed by Suchocki [48]. Appropriate amounts of gelatin (Gelia AG, Eberbach, Germany) corresponding to a given coating composition were added to the beakers (Table 4). The beakers were topped up with water. A magnetic stirrer was added to each beaker. After starting the RTC basic stirrers [IKA Poland Sp. Z o.o., Warsaw, Poland], the stirring speed was set to 400 rpm and the temperature to 60 °C. After reaching the required parameters, they were maintained for 30 min. After this time, both beakers were subjected to a cooling process until they reached 50 °C. Then, a previously measured amount of glycerol (Avantor Performance Material Poland S.A., Gliwice, Poland), equivalent to half the gelatin content, was added to each beaker. The solutions were stirred for another 30 min until the glycerol was completely dissolved at a constant stirrer speed. After this time, the coating was ready for coating the freeze-dried products.

### 4.4. Dip Coating Method

Freeze-dried bars were completely immersed in the coating solution and placed on a metal mesh to remove excess coating. Then, the coated bars were placed in an oven dryer type SUP-65 WG (Wamed, Warsaw, Poland) for 24 h at 30 °C. After drying, the bars were packed in multilayer packaging with an aluminium insert.

### 4.5. Analytical Methods

#### 4.5.1. Dry Matter Content Measurement

Approximately 1 g of coated and uncoated vegetable bars were weighed on an analytical balance into weighing vessels. The weight of the weighing vessels themselves and with the sample was recorded. The weighing vessels with the sample were placed in a WAMED SUP 65 W/G (Wamed, Warsaw, Poland) convection dryer at 60 °C for 24 h. After drying, the weighing vessels with the samples were placed in a desiccator for 30 min to cool completely and then weighed again on an analytical balance to calculate the dry matter content. The procedure was carried out in triplicate.

(1) $$d.m.=\left[\frac{\left(m_{3}-m_{1}\right)}{\left(m_{2}-m_{1}\right)}\right]*100\%$$

where *d*.*m*.—dry matter content [%]; *m*_1_—a mass of empty vessel [g]; *m*_2_—a mass of the vessel with the sample before drying [g]; and *m*_3_—a mass of the vessel with the sample after drying [g].

#### 4.5.2. Porosity Measurement

Porosity was measured using a helium pycnometer from Quantochrome Instruments (Boynton Beach, FL, USA) by the manufacturer’s instructions for use. A sample of known mass and unknown volume was placed in a pycnometer chamber of known volume, which was then tightly closed and filled with helium. The true and apparent density of the tested materials was calculated by measuring the pressure values and entering the results into the Pycnometer software version 2.7 [27].

(2) $$\varepsilon =\left(1-\frac{\rho_{d}}{\rho_{s}}\right)*100\%$$

where *ε*—porosity [%]; *ρ_d_*—real density [kg/m^3^]; and *ρ_S_*—apparent density [kg/m^3^].

#### 4.5.3. Water Activity Measurement

According to the manufacturer’s instructions, water activity was measured in a Rotronic Hydrolab C1 device (Rotronic, Bassersdorf, Switzerland). Coated and uncoated samples were placed in the device chamber. The test was performed in 3 repetitions for each type of sample.

#### 4.5.4. Real Density

This study was conducted using two 250 cm^3^ cylinders and chia seeds. Moreover, 250 cm^3^ of chia seeds were poured into the first cylinder, and a previously weighed carrot bar was placed in the second cylinder. Then, chia seeds were poured into the cylinder with the sample until the level of 250 cm^3^ was reached. The seeds remaining in the first cylinder determined the volume occupied by the sample in the second cylinder, which allowed for the calculation of the actual density [27].

(3) $$\rho =\frac{m}{V_{p}}$$

where *ρ*—sample density [g/cm^3^]; *m*—sample weight [g]; and *V_p_*—sample volume [cm^3^].

#### 4.5.5. Structure Measurement

The internal structure was examined using a scanning electron microscope TM-3000 HITACHI High-Technologies Corporation (Tokyo, Japan). Slices of 1–2 mm thickness were cut from the samples and then covered with gold to avoid excessive light penetration through the matrix. The prepared samples were placed in the measuring chamber of the microscope, where images of the surface and the centre were taken at 50× and 100× magnification.

#### 4.5.6. Determination of Sorption Isotherms

Sorption isotherms were obtained using an Aquadyne DVS analyser (Quantachrome, Boynton Beach, FL, USA), which automatically generates relative humidity and records the mass change over time. Freeze-dried bar samples (approximately 20 mg) were analysed at 25 °C, measuring moisture sorption over a relative humidity (RH) range from 1.5% to 80% with different RH change steps. The measurement was preceded by a purification step at 40 °C at 0% RH for 600 min. The relative humidity changed from one level to the next when the sample mass change was less than 0.001%/min but not earlier than 180 min from the beginning of the step [36].

Nonlinear regression analysis used Table Curve 2D v5.01 (Systat Software Inc., San Jose, CA, USA) to select the best equation for modelling the sorption isotherms. The following equations were used to describe the moisture sorption isotherms:

GAB model [37]:

(4) $$u=\frac{u_{m}Cka_{w}}{\left[(1-ka_{w})[1+(C-1)ka_{w}]\right]}$$

Halsey model [22]:

(5) $$a_{w}=exp\left(-\frac{A}{u^{B}}\right)$$

Lewicki model [41]:

(6) $$u=F\left[\frac{1}{{\left(1-a_{w}\right)}^{G}}-\frac{1}{1+a_{w}^{H}}\right]$$

Oswin model [40]:

(7) $$u=h{\left(\frac{a_{w}}{1-a_{w}}\right)}^{z}$$

Peleg model [38]:

(8) $$u=Aa_{w}^{D}+Ba_{w}^{E}$$

where *a_w_*—water activity; *u*—equilibrium water content [g H_2_O*(100 g s.s)^−1^]; *u_m_*—water content in monolayer [g H_2_O*(100 g s.s)^−1^]; and *h*, *k*, *z*, *A*, *B*, *C*, *D*, *E*, *F*, *G*, *H*—constants [37].

The fit of each model to the experimental data was assessed based on the coefficient of determination (R^2^), percentage root mean square error (*RMS*), percentage mean relative error (*MRE*), the sum of squared residuals (*RSS*), and standard error of the estimate (*SEE*).

*RMS*—relative mean square error in percentage:

(9) $$RMS=\sqrt{\frac{\sum {\left(\frac{u_{e}-u_{p}}{u_{e}}\right)}^{2}}{N}}*100\%$$

*MRE*—mean relative error in percentage:

(10) $$MRE=\frac{100}{N}*\sum \left|\frac{u_{e}-u_{p}}{u_{e}}\right|$$

*SEE*—standard error of estimate:

(11) $$SEE=\sqrt{\sum \frac{{\left(u_{e}-u_{p}\right)}^{2}}{d_{f}}}$$

*RSS*—residual sum of squares:

(12) $$RSS=\sum {\left(u_{e}-u_{p}\right)}^{2}$$

where *u_e_*—experimental equilibrium water content [g H_2_O*(100 g d.s.)^−1^]; *u_p_*—predicted equilibrium water content [g H_2_O*(100 g d.s.)^−1^]; *N*—number of measurement points; and *d_f_*—degree of freedom [18,22].

The procedure proposed by Blahovec and Yanniotis [21] was used to check the type of sorption isotherms. A plot of *aw*/*w* versus aw was obtained. and the following formula was used to describe this relationship:

(13) $$\frac{a_{w}}{w}=X_{1}+\left(X_{2}-X_{2}X_{4}\right)a_{w}-(X_{3}+X_{2}X_{4}-X_{2}X_{4}^{2})\frac{a_{w}}{1+X_{4}a_{w}}$$

where *X*_1_, *X*_2_, *X*_3_, and *X*_4_ are the equation constants, *a_w_* is the water activity, and *w* is the water content at equilibrium.

The first derivative of this function at the water activity point *a_w_* = 0 is D10. The ratio of the first derivative of the *a_w_*/*w* − *a_w_* graph in the final values to the first derivative in the initial values (*a_w_* = 0) is denoted Rfi. The extremum of the function is denoted with ‘awm’. The function’s derivatives and extremum were obtained using the Table Curve 2D programme (Systat Software Inc., San Jose, CA, USA).

#### 4.5.7. FT-IR Spectroscopy

Fourier transform infrared spectroscopy (FT-IR) of freeze-dried carrot bars was determined using the total attenuated reflection (ATR) technique with a Cary-630 spectrometer model (Agilent Technologies, Cary, NC, USA). The spectra of the analysed samples were recorded in the absorption range of 4000–650 cm^−1^ at the resolution of 4 cm^−1^. Each spectrum was an average of 32 interferograms and was presented as a dependence of the absorbance on the wavenumber.

#### 4.5.8. Statistical Methods

The obtained results were statistically analysed using a one-way analysis of variance using the Statgraphics statistical programme (https://www.statgraphics.com/).

## 5. Conclusions

Freeze-dried bars coated with pork gelatin-based coatings have the potential to be used in the food industry as products with increased durability and resistance to external factors, such as moisture or contamination. Thanks to the properties of the coatings that create a protective barrier, they can be used both as a component of dishes and as a convenient, healthy snack. An analysis of the results of tests on the effect of coating and composition of the pork gelatin coating on selected physical properties of freeze-dried carrot bars was carried out. It was shown that the coating and composition of the coating affect the change in sorption properties and selected physical parameters.

Coating freeze-dried carrot bars with coatings with the addition of 8% and 12% gelatin resulted in a significant reduction in the dry matter content and an increase in water activity compared to the control sample. The lowest dry matter content and the highest water activity were found in samples coated with a coating with the addition of 8% gelatin. These results correlate with the limited effectiveness of moisture elimination during convective drying after the coating process. A decrease in the porosity of the coated samples was also observed due to the decrease in volume compared to the control sample, which was confirmed by a microscopic examination of the internal structure. A collapse of the internal structure of the surface layer was observed, and the tightly adherent coating closed the pores of the surface layer and increased the density of pores in the centre of the sample.

Analysis of the sorption properties of freeze-dried carrot bars showed that the control sample is characterised by the highest stability in low water activity ranges, while coating with 8% gelatin significantly changes the sorption dynamics, reducing hygroscopicity in lower water activities but increasing the capillary condensation capacity in higher aw. The sample coated with 12% gelatin retains moderate hygroscopic and condensation properties, which makes it the most universal. The determined sorption isotherms are type II; the best-fit model to describe the sorption isotherms is the Peleg model. Additionally, after running the Blahovec and Yanniotis isotherm type II classification procedure, the sorption isotherms were classified as type IIb with differences in hygroscopicity and adsorption dynamics. Fourier transform infrared spectroscopy analysis did not show any significant changes in the bars’ chemical structure, and characteristic peaks were observed for individual functional groups.

## Figures and Tables

**Table 1:** The parameters of model fitting to sorption isotherms of freeze-dried carrot bars, uncoated [Control sample] and coated with 8% [8_4] and 12% [12_6] gelatin.

Parameters	Control Sample	8_4	12_6	Parameters	Control Sample	8_4	12_6
GAB				Lewicki			
u_m_	40.431546	32.62537	31.07014	F	5.496011	4.720322	4.488612
C	0.16681736	0.18998	0.190609	G	1.232432	1.355493	1.370026
k	0.85848702	0.892808	0.896351	H	3.184063	3.066408	2.943241
R^2^	0.997618	0.993505	0.995416	R^2^	0.996411	0.991468	0.993654
MRE	22.41294	77.41238	47.77109	MRE	23.92843	86.29329	54.26879
RSS	1.735079	5.374995	3.539097	RSS	2.614446	7.060499	4.899828
SEE	0.465709	0.819679	0.665122	SEE	0.571669	0.939448	0.78261
RMS [%]	42.10498	123.5829	71.01446	RMS [%]	44.19338	138.7996	80.71963
Oswin				Halsey			
z	1.107232	1.161321	1.164469	A	−4.55685	−4.67076	−4.6613
h	7.857082	7.726473	7.494353	B	−1.1535	−1.19488	−1.21622
R^2^	0.996445	0.99244	0.99455	R^2^	0.994352	0.989031	0.990766
MRE	23.45927	73.58709	45.94753	MRE	72.71449	183.8113	112.1898
RSS	2.589076	6.256256	4.207916	RSS	4.11365	9.077457	7.129626
SEE	0.568889	0.884326	0.725251	SEE	0.717082	1.065215	0.944036
RMS [%]	41.85204	115.3061	66.83861	RMS [%]	167.3527	320.5922	193.0582
Peleg							
A	29.5451	32.20775	31.42587				
B	2.877901	3.052084	3.014451				
D	29.5451	32.20775	31.42587				
E	2.89705	3.012898	3.078407				
R^2^	0.997101	0.999024	0.99906				
MRE	28.30847	22.45165	24.10866				
RSS	2.111733	0.807697	0.72588				
SEE	0.513777	0.317745	0.301223				
RMS [%]	47.72619	39.35506	42.36282				

**Table 2:** The parameters of the experimental data obtained by carrying out the procedure proposed by Blahovec and Yanniotis [21] are required to classify sorption isotherms.

Parameters	Control Sample	8_4	12_6
X_4_	4.600	4.351	4.188
R_fi_	10.578	34.359	20.570
D_10_	10.894	3.521	5.805

**Table 3:** Carrot bar composition *.

Ingredients of the Bars	Percentage Share [%]
Water	58.6
Carrot	39.8
Sodium alginate	1.5
Calcium lactate	0.1

* Own work based on Marczak [47].

**Table 4:** Symbols of freeze-dried carrot bars covered with gelatin coatings *.

Sample Symbol	Gelatin [%]	Glycerol [%]
Control	0	0
8_4	8	4
12_6	12	6

* Own work based on Ciurzyńska et al. [27].

## Data Availability

Data will be available upon reasonable request.
